# Effects of vestibular rehabilitation training combined with anti-vertigo drugs on vertigo and balance function in patients with vestibular neuronitis: a systematic review and meta-analysis

**DOI:** 10.3389/fneur.2023.1278307

**Published:** 2023-11-09

**Authors:** Jia Chen, Zhixiang Liu, Yulong Xie, Song Jin

**Affiliations:** ^1^School of Health Preservation and Rehabilitation, Chengdu University of Traditional Chinese Medicine, Chengdu, China; ^2^Rehabilitation Department, Hospital of Chengdu University of Traditional Chinese Medicine, Chengdu, China

**Keywords:** vestibular rehabilitation training, anti-vertigo drugs, vestibular neuronitis, systematic review, meta-analysis

## Abstract

**Objective:**

To investigate the effects of vestibular rehabilitation training (VRT) combined with anti-vertigo drugs on vertigo and balance function in patients with vestibular neuronitis (VN).

**Data sources:**

PubMed, EMBASE, The Cochrane Library, Web of Science, CNKI, Wan Fang Data, VIP, and CBM were searched until July 13, 2023.

**Participants:**

Patients with vestibular neuronitis participated in the study.

**Results:**

Twenty one studies including 1,415 patients were included in this review for meta-analysis. According to the Physiotherapy Evidence Database (PEDro) quality assessment, four studies received high quality (≥seven scores) and 17 studies received moderate quality (six scores). The meta-analysis showed that VRT combined with anti-vertigo drugs significantly reduced the Dizziness Handicap Inventory (DHI) score, the Vestibular Disorders Activities of Daily Living Scale (VADL) score and the Canal Paresis (CP) score, and improved the overall efficiency and the Berg Balance Scale (BBS) score, promoting vestibular evoked myogenic potentials (VEMPs) returned to normal in VN compared to simple anti-vertigo drugs or VRT alone.

**Conclusion:**

The results of this meta-analysis demonstrate the efficacy and safety of VRT combined with anti-vertigo drugs in patients with VN. Combined therapy can alleviate vestibular dysfunction such as vertigo and vomiting in patients, improve daily activity ability and balance ability, in addition to VRT has fewer adverse reactions, so it is extremely safe. However, there are shortcomings such as lack of long-term follow-up and different frequency and duration of treatment. Therefore, future randomized controlled trials (RCTs) with larger sample sizes and longer-term observations are needed to verify the effectiveness of VRT in combination with anti-vertigo drugs for VN.

**Systematic Review Registration**: https://www.crd.york.ac.uk/prospero/

## 1. Introduction

Vestibular neuritis (VN) is a common disease in otolaryngology. It is an acute unilateral vestibular dysfunction syndrome caused by inflammation of the surrounding vestibular organs (1). According to current reports, VN is the most common external vestibular disorder causing vertigo after benign paroxysmal positional vertigo (BPPV) and Meniere disease (MD) (2). The overall incidence of VN is 3.2 to 9% of all vertigo, and according to research statistics, the annual incidence of VN in Croatia is about 11.7 per 100,000 to 15.5 per 10,000 (3), mainly in middle-aged and elderly people, and the incidence of female is higher than that of male (4). The clinical manifestations of VN are acute onset, lasting more than 24 h, often accompanied by primary symptoms such as nausea, vomiting, vertigo, nystagmus, and postural instability, but without hearing impairment and central nervous system involvement (5–7), symptoms gradually resolve in most patients after a few weeks. Hypertension, diabetes, hyperlipidemia, hypothyroidism and other diseases are the common complications of VN (3). Acute symptoms such as primary vertigo and complications lead to increased physical symptoms in VN patients, negatively affecting their recovery and seriously reducing their quality of life (8).

At present, the diagnosis and treatment of VN lacks unified standards and norms, and most cases rely on exclusion methods for clinical diagnosis and treatment, which needs to be distinguished from BPPV, MD, and other diseases. And the etiology and pathogenesis of VN are unknown, but some studies have suggested that the pathogenesis may be related to the reactivation of herpes simplex virus type I in vestibular ganglia (9, 10). Some scholars have suggested that the cause of VN may be related to autoimmunity (11). Milionis’ study (12) has showed that C-reactive protein, fibrinogen, interleukin-1 (IL-1) and tumor necrosis factor α (TNF-α) in VN patients were higher than those in healthy subjects. In addition, vascular occlusion ischemia and other cardiovascular factors may also be the cause of VN (13). Xiong et al. (14) has found that serum 25-(OH)D at physiological concentration is a protective factor for VN, but low levels of serum 25-(OH)D are associated with the onset of VN. It is worth mentioning that VN is mostly unilateral and involves the superior vestibular nerve, but rarely involves the inferior vestibular nerve alone, which may be related to its anatomical structure (15).

The treatment of VN is mainly divided into drug therapy and non-drug therapy. Medications, including steroids (methylprednisolone, prednisolone, dexamethasone), antihistamine drugs (promethazine), histamines (betahistine), endogenous coenzyme b12 (mecobalamin) and alkaline drugs (sodium bicarbonate), are the most commonly used. Currently, the mechanism of drug treatment of VN mainly includes: Firstly, anti-vertigo drugs can interact with inflammatory transcription factors, thereby inhibiting pro-inflammatory molecules, reducing the number of inflammatory cells (16), effectively reducing the inflammatory response of vestibular nerve, and promoting the recovery of vestibular nerve injury. Secondly, anti-vertigo drugs can accelerate the compensation of vestibular function by the central nervous system. It has been proved in animal experiments (17, 18) that glucocorticoids can effectively promote central compensation. However, drug treatment may have some adverse effects, such as indigestion, mood swings, high blood sugar and even stomach ulcers with bleeding (19). Therefore, we must find better methods to achieve better treatment results while reducing adverse reactions.

In 1972, McCabe first proposed that VRT could reduce recurrent and prolonged vertigo (20). VRT is an exercise-based treatment that promotes the emergence of vestibular compensation by repeatedly stimulating the vestibular system (21). In 2021, “Expert Consensus on Vestibular Rehabilitation” (22), as well as other related studies (23, 24), confirmed the effectiveness and reliability of VRT for VN through clinical trials. Studies have also shown that VRT combined with drug therapy for VN may be more effective than drug therapy (25) alone or simple VRT (26).

At present, there are many clinical studies on VRT combined with anti-vertigo drugs in the treatment of VN, but there are few systematic reviews (27, 28). Among them, Hidayati et al. (27) included four studies and compared steroid drugs combined with VRT with steroid drugs or VRT alone. However, the main content was the difference in efficacy between steroid drugs and VRT. The review finally concluded that there was no difference in long-term efficacy between the two. And whether to combine steroid drugs with VRT is an issue that needs to be considered. Most recently, Huang et al. (28) compared steroid drugs combined with VRT as an intervention method in the experimental group with steroid drugs in the control group, and the outcome indicators included DHI score, caloric lateralization and VEMPs. It was concluded that steroid drugs combined with VRT was more effective than steroid alone. However, there are few included studies and outcome indicators. There are many drugs currently used to treat VN, not just steroids. In addition, many clinical studies have shown that when VN patients feel vertigo, their balance ability and daily activities will also be greatly affected. We believe that this is the biggest worry and annoyance of VN patients, and it is also a priority problem for patients to solve during treatment. However, these two related meta-analyses only considered steroid medications and did not focus on relevant outcome measures such as balance and daily activities. Based on the above reviews, the goal of our meta-analysis was to analyze a large number of studies, so we developed inclusion criteria from different perspectives, expanded the sample size, and finally included 21 RCTs with a total of 1,425 subjects. In terms of outcome measurement, we included six outcome indicators, including DHI score, VADL score, CP score, BBS score, overall efficiency and VEMPs, to provide a more comprehensive analysis of results. The aim is to update and expand the efficacy and safety of VRT combined with anti-vertigo drugs in the treatment of VN, and to provide a more reliable basis for the follow-up clinical research.

## 2. Materials and methods

### 2.1. Search strategy

**Figure 1:**
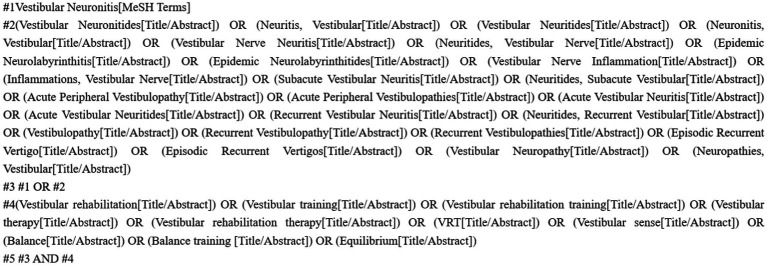
Pubmed search history.

This review was reported in conformity to the Preferred Reporting Item for Systematic Review and Meta-analyses Statement. We searched PubMed, EMBASE, Web of science, the Cochrane Library, China National Knowledge Infrastructure (CNKI), Technology Periodical Database (VIP), Wan Fang Data, and China Biology Medicine (CBM) from the earliest available date until July 13, 2023. Chinese search keywords included “#1 vestibular neuronitis/vestibular nerve inflammation/acute peripheral vestibulopathy/episodic recurrent vertigo/vestibular neuropathy”; #2 “vestibular rehabilitation training/vestibular rehabilitation/vestibular training/vestibular therapy/VRT/balance training.” English search was conducted using keywords and their varies (Figure 1), including “vestibular neuronitis” and “vestibular rehabilitation training.” The language was restricted to Chinese and English and the study type was only required to be a randomized controlled trial (RCT).

### 2.2. Inclusion criteria

After we reviewed relevant articles, eligibility criteria for this review based on PICOS frameworks (population, intervention, comparison, outcome, and study) were as follows: (1) Participants: RCTs of patients with VN which published in English or Chinese. Differences in sex, age, country, time, and race were not taken into account. (2) Intervention: VRT combined with anti-vertigo drugs (such as methylprednisolone, betahistine mesilate, dexamethasone sodium phosphate, promethazine, sodium bicarbonate, mecobalamin) administered to patients. (3) Comparison: Anti-vertigo drugs or VRT as a control intervention. (4) Outcome: At least one outcome index such as overall efficiency, Dizziness Handicap Inventory (DHI), Berg Balance Scale (BBS), Vestibular Evoked Myogenic Potentials (VEMPs), Vestibular Disorders Activities of Daily Living Scale (VADL), and Canal Paresis (CP) score.

### 2.3. Exclusion criteria

(1) Non-RCTs. (2) Test number ≤ 10 (Because fewer subjects may lead to inaccurate results. Here 10 represents 10 subjects in each group). (3) Unable to get full text or incomplete article data. (4) Both intervention methods were VRT.

### 2.4. Data extraction

Two authors (J.C. and Y.L.X.) screened studies according to inclusion and exclusion criteria and collected data independently. Information such as author name, year of publication, age of patients in the trial and control groups, sample size, intervention mode, treatment frequency, duration, and outcome were recorded. All studies are managed using Endnote X9. Differences are resolved by discussion or arbitration by a third reviewer (Z.X.L.).

### 2.5. Quality assessment

We assessed the quality of the literature using the Physicaltherapy Evidence Database (PEDro). The Pedro scale uses 11 criteria, each of which is rated “yes” or “no,” with one point awarded for each response. The first item does not count toward the PEDro score, which is a total of 10 points. PEDro total score ≥ seven points is classified as high quality, five to six points is classified as medium quality, ≤ four points is classified as low quality. The scores were given independently by two reviewers (J.C. and Y.L.X.). If the results are inconsistent, they are discussed with a third reviewer (Z.X.L.).

Two reviewers (J.C. and Y.L.X.) also completed the risk of bias. The evaluation was based on the Cochrane Handbook for Systematic Review of Interventions, edition 5.3. Items include: (1) random sequence generation (selection bias). (2) allocation concealment (selection bias). (3) blinding of participants and personnel (performance bias). (4) blinding of outcome assessment (detection bias). (5) incomplete outcome data (attrition bias). (6) selective reporting (reporting bias). (7) other bias. The quality of the included studies was rated as low/unclear/high risk of bias (low risk of bias as “yes,” high risk of bias as “no,” otherwise was “unclear”).

### 2.6. Statistical analysis

We developed inclusion/exclusion criteria for screening articles, followed by data extraction and quality assessment. We used StataMP 14.0 software to conduct meta-analysis and give the final results. For continuous data, mean difference (MD) and 95% confidence intervals (CI) were used when evaluating results using the same scale. Two statistical tests were used to assess inter-study heterogeneity. If *I*^2^ < 50% or *p* > 0.05, it was considered low heterogeneity, and the fixed effects model was used to merge the data. If *I*^2^ > 50% or *p* < 0.05 implies high heterogeneity, a random effects model was used for meta-analysis and subgroup analysis or sensitivity analysis was considered to determine the source of heterogeneity. Overall efficiency was classified into two levels: (1) effective and (2) ineffective. Overall efficiency referred to the percentage of participants in the first two levels as a percentage of the total. Publication bias was studied by funnel plot and Egger’s test was used to verify the bias of the funnel plot.

### 2.7. Trial sequential analysis

Meta-analyses often require multiple tests, and random errors can sometimes lead to false significance results when accumulating data, and the increased frequency of statistical tests in meta-analyses increases the likelihood of reporting such results. However, trial sequence analysis (TSA) overcomes the shortcomings of classical meta-analysis and corrects for the increase in type I errors.

Sequence analysis was performed using TSA.0.9.5.10 beta. If the Z-curve exceeds the traditional boundary, but does not cross the TSA boundary, it indicates a possible false positive error. If it intersects the TSA boundary, it indicates that the meta-analysis results are robust, even if RIS is not reached. The Z-curve does not intersect the traditional cutoff values and the TSA cutoff values, and a positive or negative conclusion cannot be drawn. The Z-curve intersects the zero line, indicating no significance. We set a 5% risk of type I error (α) and a 20% risk of type II error (β) to calculate the amount of information required, and reduced the relative risk (RRR) and control event rate by 20% based on the data from the meta-analysis.

## 3. Result

### 3.1. Selection and inclusion of studies

**Figure 2:**
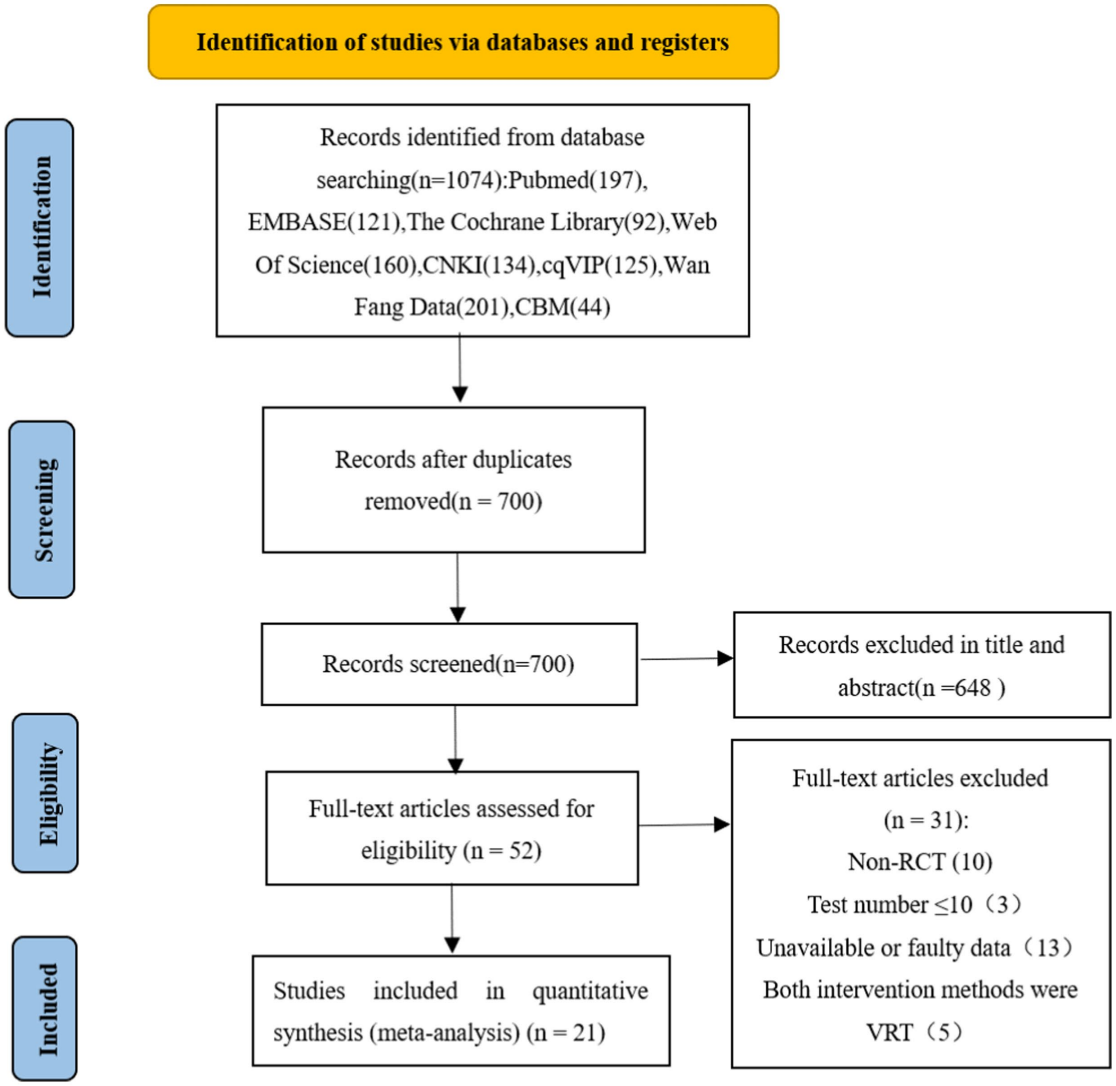
Flowchart of the selection process.

A total of 1,074 studies were initially screened (PubMed = 197, EMBASE = 121, The Cochrane Library = 92, Web of Science = 160, CNKI = 134, Wan Fang Data = 201, Vip = 125, CBM = 44). After primary searches from the databases, 700 articles were screened. After duplicates removed, reading the titles and abstracts, 648 articles were excluded. Full texts of 52 articles were retrieved, and 31 articles were excluded with reasons listed as the following: non-RCT (*n* = 10), test number ≤ 10 (*n* = 3), unavailable or faulty data (*n* = 13) and both intervention methods were VRT (*n* = 5). In the end, 21 RCTs were included. Three were written in English, 18 of which were written in Chinese. The detailed screening process was shown in Figure 2.

### 3.2. Characteristics of included studies

A total of 21 studies (25, 26, 29–47) involving 8 datasets were included. All of the studies were published between 2014 and 2023 in English or Chinese. The sample size ranged from 29 to 200. All experimental groups received VRT combined with anti-vertigo drugs. Among them, anti-vertigo drugs included methylprednisolone, dexamethasone, betahistine, sodium bicarbonate, mecobalamin, promethazine. The control groups underwent VRT or antivertigo therapy as did the experimental group. The primary outcomes included the overall efficiency, DHI score and BBS. The secondary outcomes included VEMPs, CP score, and VADL score. Characteristics of these studies are shown in Table 1. There was no significant difference in baseline data between the two groups.

**Table 1:** Characteristics summary of included studies.

Study	Country	Experimental group				Control group				Drug dose and number of days	duration	Outcomes	Positive/negative
		Age	Sample size	Intervention	Frequency	Age	Sample size	Intervention	Frequency				
Chen (40)	China	42.49 ± 1.25	19	VRT+ Methylprednisolone, Betahistine mesilate	2/day	42.52 ± 1.21	19	Methylprednisolone, Betahistine mesilate	1/day	20–80 mg/d, 36 mg/d, 9 days	9 days	B + E	+
Fan et al. (39)	China	37.2 ± 13.1	30	VRT + Prednisolone, Betahistine	3/day	36.6 ± 11.9	30	Prednisolone, Betahistine	3/day	1 mg/kg, 12 mg/d, NR	24 weeks	B + F	+
Goudakos et al. (25)	Greece	53.95	20	VRT+ Dexamethasone sodium phosphate	1/day	51.75	20	Dexamethasone sodium phosphate	1/day	24 mg/d, 14 days	2 weeks	B + D	+
Ismail et al. (29)	Egypt	49.1 ± 12.8	20	VRT + Methylprednisolone	1/day	47.9 ± 13.9	20	Methylprednisolone	3/day	60 mg/d, 2 weeks	6 weeks	B + D	+
Ismail et al. (29)	Egypt	49.1 ± 12.8	20	VRT + Methylprednisolone	1/day	49.3 ± 11.6	20	VRT	1/day	60 mg/d, 2 weeks	6 weeks	B + D	+
Li et al. (26)	China	41.36 ± 5.92	35	VRT + Prednisone	3/day	42.01 ± 5.64	35	VRT	2/day	30 mg/d, 4 weeks	4 weeks	A + C	+
Li (43)	China	40.48 ± 2.05	43	VRT+ Sodium Bicarbonate	2/day	41.03 ± 3.28	43	Sodium Bicarbonate	1-2/day	40 mL/d, 3–5 days	3–5 days	B + C	+
Liao et al. (47)	China	42.53 ± 10.15	15	VRT + Promethazine, Betahistine	2/day	43.6 ± 10.75	15	Promethazine, Betahistine	3/day	30 mg/d, 36 mg/d, NR	4 weeks	B	+
Liu et al. (42)	China	41.30 ± 5.11	25	VRT+ Betahistine, Methylprednisolone	1/day	40.18 ± 5.03	25	Betahistine, Methylprednisolone	3/day	36 mg/d, 20–80 mg/d, 2 weeks	NR	A + B + E	+
Lu et al. (38)	China	56.2 ± 0.8	25	VRT+ Betahistine, Methylprednisolone	1/day	55.6 ± 0.5	25	Betahistine, Methylprednisolone	3/day	36 mg/d, 20–80 mg/d, 2 weeks	2 weeks	A + B + E	+
Shen et al. (41)	China	45.2 ± 3.6	100	VRT+ Dexamethasone, Prednisone, mecobalamin	≥3/day	43.5 ± 2.7	100	Dexamethasone, Prednisone, mecobalamin	3/day	NR	NR	A	+
Wang et al. (45)	China	19–73	26	VRT+ Methylprednisolone	≥2/day	19–73	24	Methylprednisolone	3/day	20–80 mg/d, 9 days	4 weeks	D + F	+
Wang et al. (31)	China	41.30 ± 6.25	35	VRT+ Betahistine, Prednisone	3/day	41.26 ± 6.38	35	Betahistine, Prednisone	3/day, 1/day	36 mg/d, 30 mg/d, 5 days	4 weeks	B + F	+
Wu (33)	China	39.17 ± 4.25	32	VRT+ Betahistine, Prednisone	NR	38.46 ± 3.79	32	Betahistine, Prednisone	3/day	18 mg/d, 1 mg/kg, NR	NR	B + C + E	+
Xu et al. (32)	China	47.3 ± 3.4	50	VRT+ Methylprednisolone	2-3/day	48.5 ± 3.5	50	Methylprednisolone	NR	20-80 mg/d, 9 days	4 weeks	A + B + E	+
Yan et al. (46)	China	47.8 ± 2.0	28	VRT+ Betahistine, Prednisone	3/day	49.2 ± 1.6	20	Betahistine, Prednisone	3/day	36 mg/d, 1 mg/kg, NR	2 weeks	A + B	+
Yan et al. (44)	China	46.58 ± 9.71	72	VRT+ mecobalamin, etc	3/day	46.58 ± 9.71	72	mecobalamin, etc.	3/day	1.5 mg/d, 10 days	10 days	A	+
Yoo et al. (30)	Korea	54.1 ± 12.5	15	VRT+ Methylprednisolone+ *Ginkgo biloba* extract	≤10/day	59.6 ± 11.8	14	*Ginkgo biloba* extract	2/day	80 mg/d, 14 days	4 weeks	B	+
Zhang et al. (34)	China	35.6 ± 10.1	29	VRT + Promethazine, Betahistine	2/day	35.8 ± 10.2	29	Promethazine, Betahistine	3/day	30 mg/d, 36 mg/d, 4 weeks	4 weeks	A + B + E	+
Zhao et al. (37)	China	39.72 ± 9.41	40	VRT+ Betahistine, Dexamethasone	3/day	39.43 ± 9.50	40	Betahistine, Dexamethasone	3/day, 1/day	48 mg/d, 10–20 mg/d, 7 days	12 weeks	A + B + C	+
Zhao (35)	China	40.35 ± 3.18	27	VRT+ Betahistine, Dexamethasone	3/day	40.25 ± 3.21	26	Betahistine, Dexamethasone	3/day, 1/day	48 mg/d, 10–20 mg/d, 7 days	12 weeks	A + B + C	+
Zhao et al. (36)	China	47.49 ± 8.92	23	VRT+ Betahistine, Dexamethasone, Promethazine	2/day	47.95 ± 9.04	22	Betahistine, Dexamethasone, Promethazine	3/day	36 mg/d, 2 mg/d,25 mg/d, 2 weeks	4 weeks	B	+

A, Overall efficiency; B, DHI, Dizziness Handicap Inventory; C, BBS, Berg Balance Scale; D, VEMPs, Vestibular Evoked Myogenic Potentials (VEMPs); E, VADL, Vestibular Disorders Activities of Daily Living Scale; F, CP score, Canal Paresis score; VRT, vestibular rehabilitation training; NR: Not Reported.

### 3.3. Methodological quality of included studies

The quality of the included studies was evaluated according to the PEDro quality assessment scale, most of all had methodological deficiencies in the blinding of subjects, therapists, and assessors. Four studies obtained high quality and 17 studies obtained moderate quality, as detailed in Table 2.

**Table 2:** Evaluation of the quality of the included documents through PEDro.

Study	1	2	3	4	5	6	7	8	9	10	11	Total score	Level
Chen (40)	**√**	**√**	**×**	**√**	**×**	**×**	**×**	**√**	**√**	**√**	**√**	6	Medium
Fan et al. (39)	**√**	**√**	**×**	**√**	**×**	**×**	**×**	**√**	**√**	**√**	**√**	6	Medium
Goudakos et al. (25)	**√**	**√**	**√**	**√**	**×**	**×**	**√**	**√**	**√**	**√**	**√**	8	High
Ismail et al. (29)	**√**	**√**	**×**	**√**	**×**	**×**	**√**	**√**	**√**	**√**	**√**	7	High
Li et al. (26)	**√**	**√**	**×**	**√**	**×**	**×**	**×**	**√**	**√**	**√**	**√**	6	Medium
Li (43)	**√**	**√**	**×**	**√**	**×**	**×**	**×**	**√**	**√**	**√**	**√**	6	Medium
Liao et al. (47)	**√**	**√**	**×**	**√**	**×**	**×**	**×**	**√**	**√**	**√**	**√**	6	Medium
Liu et al. (42)	**√**	**√**	**×**	**√**	**×**	**×**	**×**	**√**	**√**	**√**	**√**	6	Medium
Lu et al. (38)	**√**	**√**	**×**	**√**	**×**	**×**	**×**	**√**	**√**	**√**	**√**	6	Medium
Shen et al. (41)	**√**	**√**	**×**	**√**	**×**	**×**	**×**	**√**	**√**	**√**	**√**	6	Medium
Wang et al. (45)	**√**	**√**	**×**	**√**	**×**	**×**	**×**	**√**	**√**	**√**	**√**	6	Medium
Wang et al. (31)	**√**	**√**	**×**	**√**	**×**	**×**	**×**	**√**	**√**	**√**	**√**	6	Medium
Wu (33)	**√**	**√**	**×**	**√**	**×**	**×**	**×**	**√**	**√**	**√**	**√**	6	Medium
Xu et al. (32)	**√**	**√**	**×**	**√**	**×**	**×**	**×**	**√**	**√**	**√**	**√**	6	Medium
Yan et al. (46)	**√**	**√**	**×**	**√**	**×**	**×**	**×**	**√**	**√**	**√**	**√**	6	Medium
Yan et al. (44)	**√**	**√**	**×**	**√**	**×**	**×**	**×**	**√**	**√**	**√**	**√**	6	Medium
Yoo et al. (30)	**√**	**√**	**√**	**√**	**×**	**×**	**×**	**√**	**√**	**√**	**√**	7	High
Zhang et al. (34)	**√**	**√**	**×**	**√**	**×**	**×**	**×**	**√**	**√**	**√**	**√**	6	Medium
Zhao et al. (37)	**√**	**√**	**×**	**√**	**×**	**×**	**×**	**√**	**√**	**√**	**√**	6	Medium
Zhao (35)	**√**	**√**	**×**	**√**	**×**	**×**	**×**	**√**	**√**	**√**	**√**	6	Medium
Zhao et al. (36)	**√**	**√**	**√**	**√**	**×**	**×**	**×**	**√**	**√**	**√**	**√**	7	High

1 = inclusion exclusion criteria; 2 = randomized group; 3 = allocation concealment; 4 = similar baseline; 5 = subject blinding; 6 = therapist blinding; 7 = assessor blinding; 8 = more than 85% of patient measures; 9 = intention to treat; 10 = between-group analysis; 11 = at least one point measure (√: yes, no risk; ×: no, risky).

### 3.4. Risk of bias in studies

**Figure 3:**
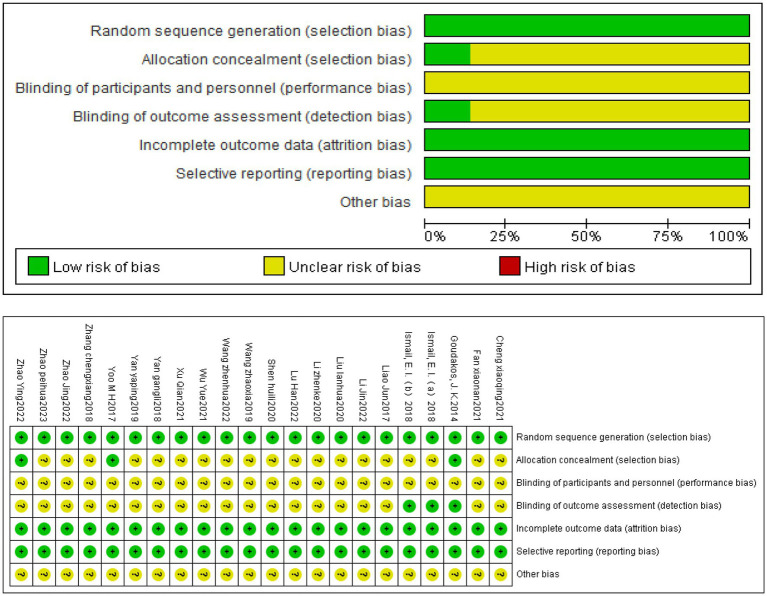
Risk of bias of included studies.

The plot of the risk of bias for each included study are shown in Figure 3, whole trials are at low risk. All of 21 studies reported random sequence generation and were assessed as low risk. Eighteen studies were assessed as unclear risk, and 3 were assessed as low risk in the aspect of allocation concealment. In blinding of participants and personnel, total studies were assessed as unclear risk because of no report. What is more, 18 studies were assessed as unclear risk and 3 studies were assessed as low risk in the blinding of the outcome assessment. Of all these 21 studies were judged to be low risk in incomplete outcome data and selective reporting. Finally, 21 studies were assessed as unclear risk in other bias.

### 3.5. Publication bias

**Figure 4:**
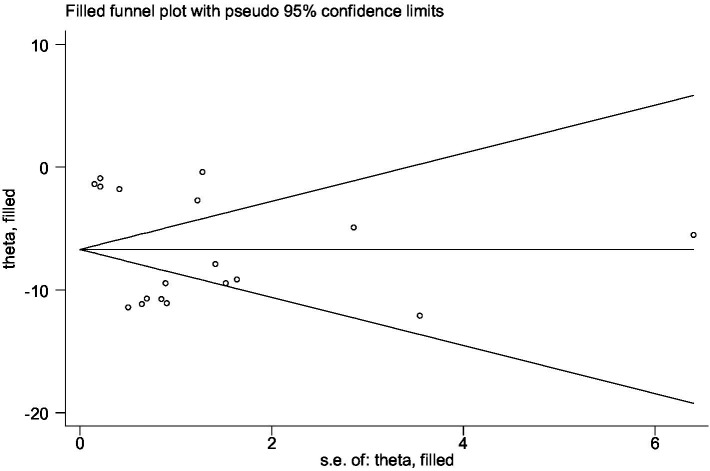
Trim and fill analysis of publication bias by DHI score.

We used StataMP 14 to conduct publication bias analysis of Egger’s test for DHI scores with RCTs >10. The Egger’ test result showed *p* < 0.05, which might lead to publication bias, so it was corrected by the trim and fill analysis. The correction result is shown in Figure 4. There was no significant difference between the corrected result and the pre-corrected result, which proved that the funnel plot was basically symmetric and the results of this meta-analysis were stable without publication bias. In addition, most of the studies in this meta-analysis are from China, and although most of them have good correlation and reliability, it may still lead to national publication bias, which is a problem worthy of attention and needs to be solved in the future.

### 3.6. Trial sequential analysis

**Figure 5:**
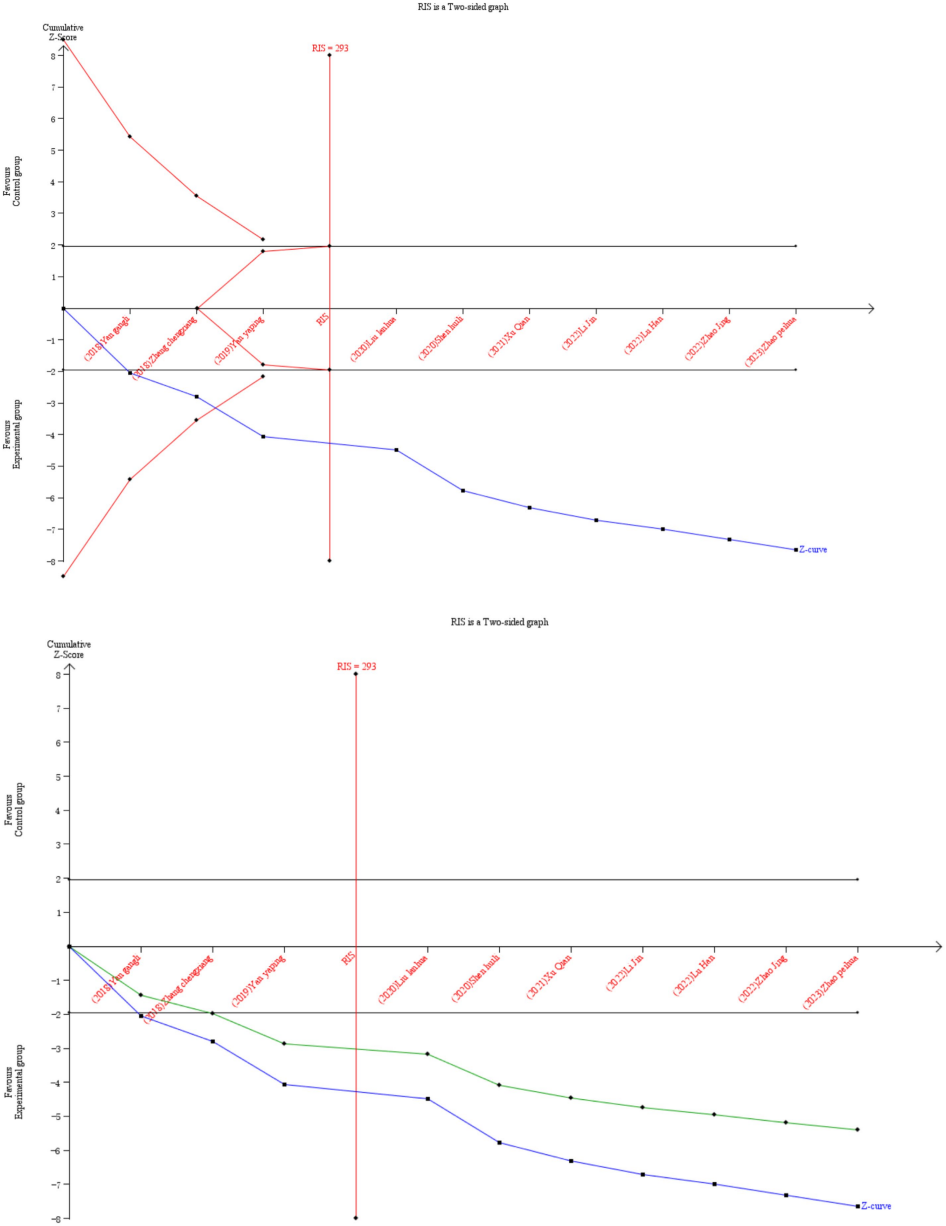
Comparison of overall efficiency of VRT combined with antivertigo drugs in the treatment of VN. The black line represents the conventional statistical boundary at *p* = 0.05. The blue line represents the cumulative Z-score of the meta-analysis. Red lines indicate TSA boundaries. The green line shows the Z-curve after penalty statistics. RIS represents the amount of information required.

Ten RCTs (26, 32, 34, 35, 37, 38, 41, 42, 44, 46) reported the overall efficiency of the binary variable, which were analyzed sequentially, with a type I error of 5% and a statistical power of 80%. The information axis was set as the cumulative sample size, and the sample size was used as the expected information value (RIS). Figure 5 shows that the Z-curve crosses the conventional boundary value and the TSA boundary value, indicating that the results obtained from this meta-analysis are robust and the efficacy of VRT combined with anti-vertigo drugs in the treatment of VN is positive. Meantime, the penalty curve also exceeded the traditional boundary value and reached the RIS value, which made the meta-analysis result more stable.

### 3.7. Meta-analysis results

#### 3.7.1. Primary outcomes

##### 3.7.1.1. Result of the overall efficiency

**Figure 6:**
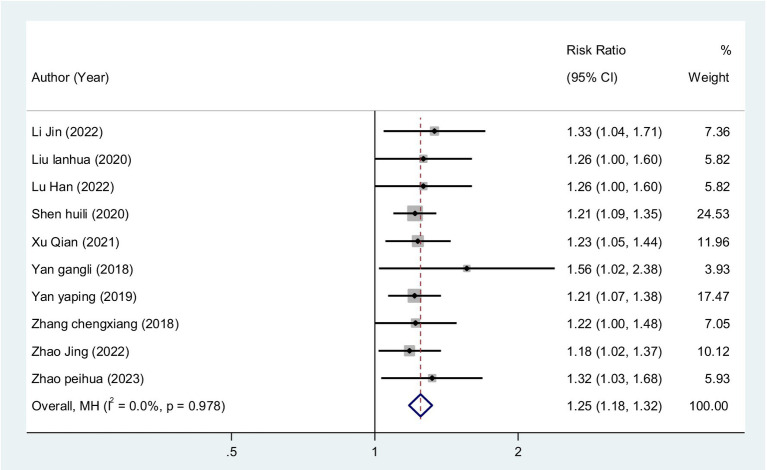
Forest plot of overall efficiency.

A total of 10 studies (26, 32, 34, 35, 37, 38, 41, 42, 44, 46) evaluated 853 participants and reported overall efficiency. Data were pooled using a fixed effect model (*I*^2^ = 0%, *p* = 0.978 > 0.05) (Figure 6), and the result showed that VRT combined with anti-vertigo drugs was adequate for the treatment of VN compared with the control group [RR = 1.25, 95% CI (1.18, 1.32)].

##### 3.7.1.2. Result of the DHI score

**Figure 7:**
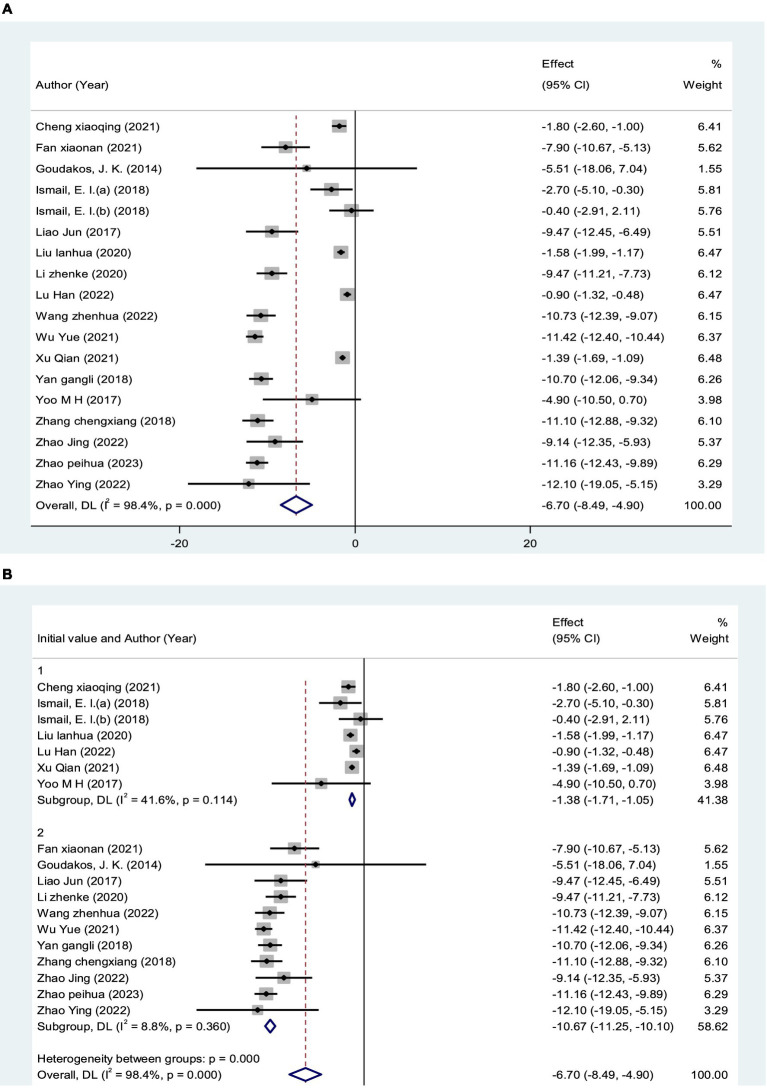
Forest plot of DHI score. **(A)** All studies. **(B)** After subgroup analysis (15 points indicates the initial DHI score).

A total of 18 RCTs (25, 29–40, 42, 43, 46, 47), including 981 patients, reported DHI scores. The DHI scores of 18 studies were analyzed, showing statistical heterogeneity among the studies (*I*^2^ = 98.4%, *p* < 0.0001). The random effect model was used for meta-analysis. The results showed that the treatment effect of the experimental group was better than the control group [MD = −6.70, 95% CI (−8.49, −4.90)] (Figure 7A), which could prove that VRT combined with anti-vertigo drugs had a positive effect on relieving the degree of vertigo in VN patients. Because of the significant heterogeneity of DHI score, a subgroup analysis of initial DHI score (<15 points or ≥15 points) of VN patients showed that heterogeneity was reduced in both groups. We found that 7 RCTs with a total of 347 VN patients had an initial DHI score of <15 points, and VRT combined with anti-vertigo drugs significantly reduced DHI score compared with the control group[MD = −1.38, 95% CI (−1.71, −1.05), *I*^2^ = 41.6%, *p* = 0.114 > 0.05]. The initial DHI score of 634 VN patients in 11 RCTs was ≥15 points, and the results also showed that the combined group could better relieve the vertigo state of VN patients and reduce the DHI score [MD = −10.67, 95% CI (−11.25, −10.10), *I*^2^ = 8.8%, *p* = 0.360>0.05]. The results of subgroup analysis proved that VRT combined with anti-vertigo drugs had positive significance in improving the symptoms of patients. In addition, we can infer from the results that the higher the initial DHI score, the more significant the reduction of vertigo after treatment (Figure 7B).

##### 3.7.1.3. Result of the BBS score

**Figure 8:**
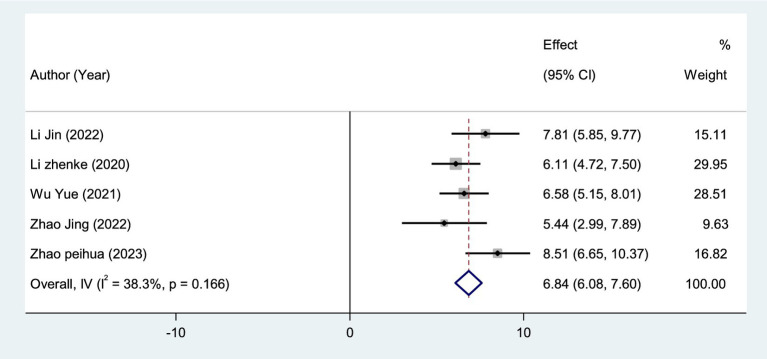
Forest plot of BBS.

A total of 5 studies (26, 33, 35, 37, 43) assessed 398 participants who reported BBS scores. We used a fixed-effect model (*I*^2^ = 38.3%, *p* = 0.116 > 0.05) to aggregate the data (Figure 8), and the results showed that VRT combined with anti-vertigo drugs could better enhance the balance function of VN patients compared with the control group [MD = 6.84, 95%CI (6.08, 7.60)].

#### 3.7.2. The secondary outcomes

##### 3.7.2.1. Result of the VEMPs

**Figure 9:**
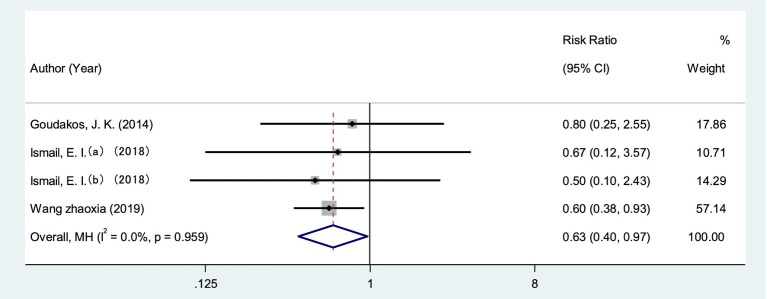
Forest plot of VEMPs.

A total of 3 studies (one three-arm study) (25, 29, 45) measured VEMPS in 154 patients. The fixed effect model showed statistical significance compared with the control group (*I*^2^ = 0%, *p* = 0.959 > 0.05), indicating that VRT combined with anti-vertigo drugs can improve vestibular muscle and nerve function in VN patients [RR = 0.63, 95% CI (0.40, 0.97)] (Figure 9).

##### 3.7.2.2. Result of the CP score

**Figure 10:**
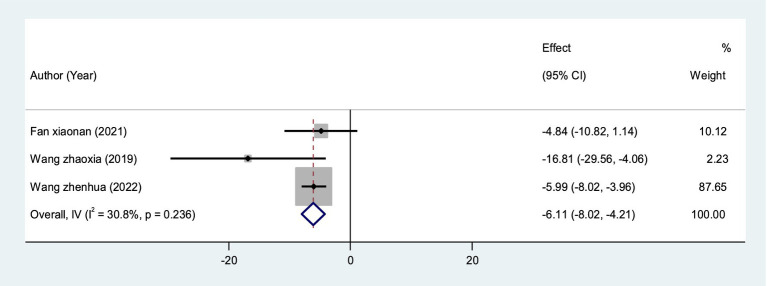
Forest plot of CP score.

A total of 3 RCTs (31, 39, 45) measured CP score values in 180 patients. The fixed-effect model showed statistical significance compared with the control group (*I*^2^ = 30.8%, *p* = 0.236 > 0.05), indicating that VRT combined with anti-vertigo drugs could significantly reduce canal paresis in VN patients [MD = −6.11, 95%CI (−8.02, −4.21)] (Figure 10).

##### 3.7.2.3. Result of the VADL score

**Figure 11:**
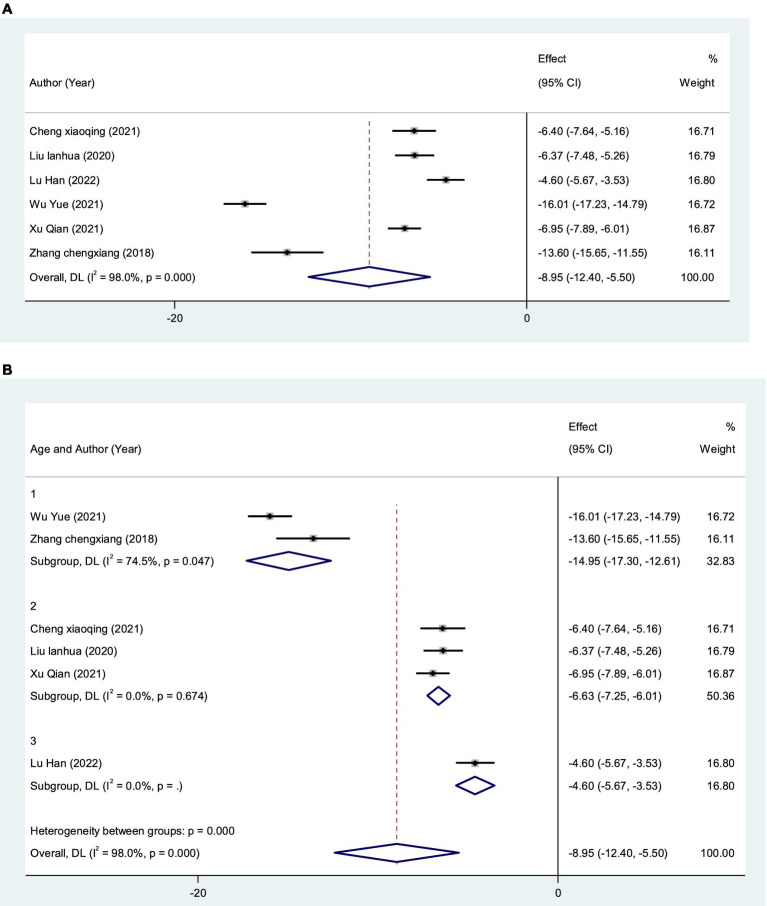
Forest plot of VADL score. **(A)** All studies. **(B)** After subgroup analysis.

A meta-analysis of the VADL scores of 360 VN patients from 6 studies (32–34, 38, 40, 42) was performed using a random-effects model. The VADL score of VRT combined with anti-vertigo drugs was significantly lower than that of control group [MD = −8.95, 95%CI (−12.39, −5.52), *I*^2^ = 98.0%, *p* < 0.001] (Figure 11A). Due to the significant heterogeneity, we performed a subgroup stratified analysis on the age of VN patients (<40 years, 40–50 years old, >50 years) to reduce heterogeneity. Using the random effects model, the results showed the first group [MD = −14.95, 95%CI (−17.30, −12.61), *I*^2^ = 74.5%, *p* = 0.047], the second group [MD = −6.63, 95%CI (−7.25, −6.01), *I*^2^ = 0%, *p* = 0.674 > 0.05], and the third group [MD = −4.60, 95%CI (−5.67, −3.53), *p* < 0.05]. This suggested that VRT combined with anti-vertigo drugs could significantly reduce VADL scores in VN patients, thereby improving their daily activities and vestibular function (Figure 11B).

### 3.8. Sensitivity analysis

**Figure 12:**
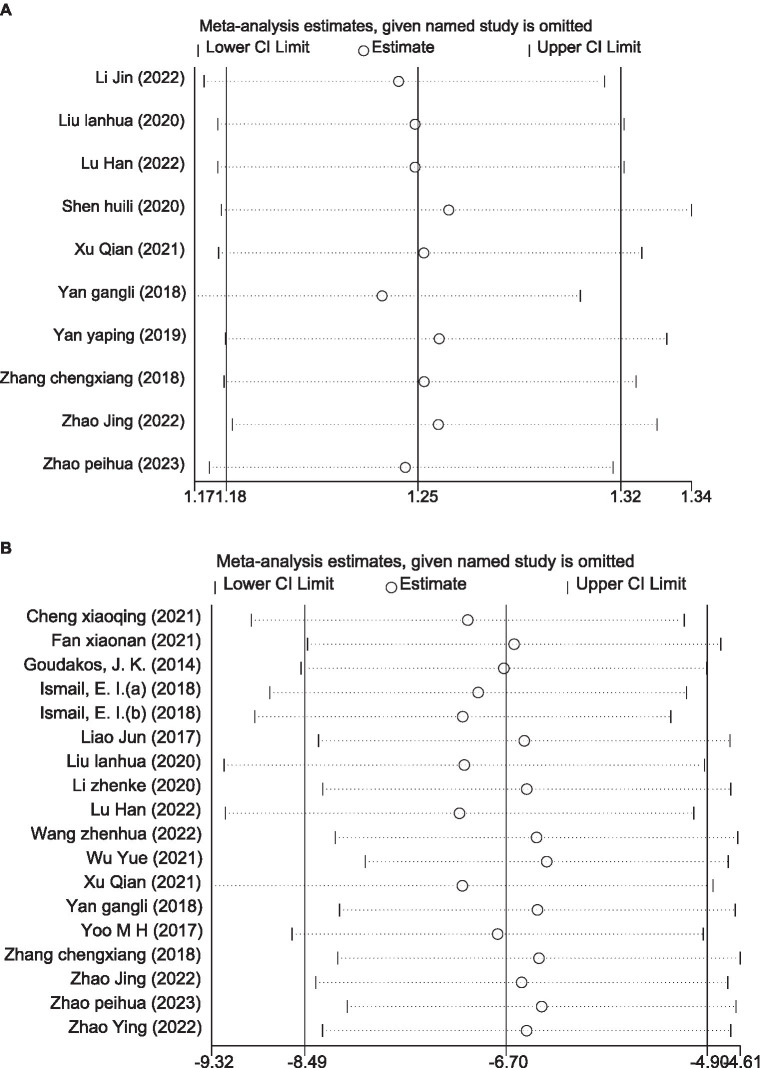
Result of sensitivity analysis. **(A)** overall efficiency. **(B)** DHI.

We used StataMP 14. for sensitivity analysis of the results. Firstly, the overall efficiency results are shown in Figure 12A. The meta-analysis included 10 studies, and the pooled results found that removing any of the articles did not have a strong effect on the results. The results were consistent with the meta-analysis [RR = 1.25, 95% CI (1.18, 1.32)], which proved that the meta-results were stable. The second was the sensitivity analysis of DHI, with a total of 18 RCTs, which was also found to be consistent with the meta-analysis [MD = −6.70, 95% CI (−8.49, −4.90)], indicating that the meta-results were stable (Figure 12B).

### 3.9. Meta-regression results

Due to the significant heterogeneity in the meta-analysis results of DHI data (*I*^2^ = 98.4%), based on the included RCTs, we performed meta regression on the age of VN patients, country, initial DHI score and type of anti-vertigo drugs to find the source of heterogeneity. The results showed that the age of VN patients (*p* = 0.031 < 0.05) and DHI initial score (*p* < 0.001) were the sources of heterogeneity (Table 3), while the country (*p* = 0.895 > 0.05) and the type of anti-vertigo drugs (*p* = 0.411 > 0.05) were not the source of heterogeneity. Similarly, there was significant heterogeneity in the meta-analysis of VADL (*I*^2^ = 98.0%). We performed meta regression on the age of VN patients and the type of anti-vertigo drugs, and found that the age of VN patients (*p* = 0.027 < 0.05) was the source of heterogeneity, while the type of anti-vertigo drugs (*p* = 0.638 > 0.05) was not the source of heterogeneity (Table 3).

**Table 3:** Meta-regression results of DHI and VADL.

Outcome	_ES	Coef.	Std. Err.	*t*	*p*>|*t*|	[95% Conf. Interval]	
DHI	Age	0.6378277	0.2643176	2.41	0.031	0.0668042	1.208851
	Country	−0.1262971	0.9419664	−0.13	0.895	−2.161292	1.908698
	Initial score	−8.874486	0.3828021	−23.18	0.000	−9.70148	−8.047493
	Drug type	−0.0516424	0.0607738	−0.85	0.411	−0.1829361	0.0796514
	_cons	6.385251	1.264856	5.05	0.000	3.652696	9.117806
VADL	Age	5.827061	1.427994	4.08	0.027	1.282548	10.37157
	Drug type	−0.3031553	0.5816561	−0.52	0.638	−2.154245	1.547934
	_cons	−18.50436	3.1693	−5.84	0.010	−28.59049	−8.418235

## 4. Discussion

This meta-analysis included 21 studies with 1,425 VN patients. The experimental groups were treated with VRT combined with anti-vertigo drugs (such as methylprednisolone, betahistine mesilate, dexamethasone sodium phosphate, promethazine, sodium bicarbonate, mecobalamin) and the control groups received the same antivertigo drug or VRT (Table 1). To assess the quality of the included studies, we used the PEDro scale, which assessed 4 of 21 studies as high quality and 17 of medium quality. For the assessment of the risk of bias, 21 studies all described randomization methods and reported primary outcome measures. However, because other bias assessment risks were not reported in the studies, the risk assessment for bias was not known, and ultimately, the risk assessment for bias was evaluated as low. Egger’ test was adopted to analyze the publication bias of DHI score, and the result showed that DHI score may lead to publication bias (*p* < 0.05). We further corrected it by trim and fill analysis, and the result showed that no new literature was added on the funnel plot. This proved that there was no significant difference from the results before correction, and the funnel plot was basically symmetric, that is, the results of this meta-analysis were stable without publication bias. In addition, we also used TSA to conduct stability tests on the results of the overall efficiency, and the results showed that the meta-analysis of the overall response rate was robust.

Meta-analysis results demonstrated that VRT combined with anti-vertigo drugs could reduce DHI score in VN patients compared with the control group [MD = −6.70, 95% CI (−8.49, −4.90), *I*^2^ = 98.4%, *p* < 0.0001]. Obviously, there was significant heterogeneity in this meta-analysis. To identify the source of heterogeneity, we performed meta regression according to the age of VN patients, initial DHI score and type of anti-vertigo drugs. Subgroup analysis based on initial DHI score (<15 points or ≥15 points) showed that [MD = −1.38, 95% CI (−1.71, −1.05), *I*^2^ = 41.6%, *p* = 0.114 > 0.05] (initial DHI score < 15 points) and [MD = −10.67, 95% CI (−11.25, −10.10), *I*^2^ = 8.8%, *p* = 0.360 > 0.05] (initial DHI score ≥ 15 points). The reason why we are making sub-group analysis for initial DHI score is because in the process of data entry, two reviewers have found that there is a wide difference in initial DHI score for different articles, which is highly likely to lead to significant heterogeneity. The results showed that VRT combined with antivertigo had positive effect on improving vertigo state in VN patients. In order to further verify the stability of the meta results, we conducted a sensitivity analysis and found that no single article had a strong impact on the results, which was consistent with the original combined results [MD = −6.70, 95% CI (−8.49, −4.90)], and the results were stable. We adopted the same analysis method for VADL with significant heterogeneity [MD = −8.95, 95%CI (−12.39, −5.52), *I*^2^ = 98.0%, *p* < 0.001]. We performed meta regression according to the age of VN patients and type of anti-vertigo drugs. Subgroup analysis based on the age of VN patients (<40 years old, 40–50 years old, >50 years old) showed that [MD = −14.95, 95%CI (−17.30, −12.61), *I*^2^ = 74.5%, *p* = 0.047] (age < 40 years old), [MD = 6.63, 95% CI (7.25, 6.01), *I*^2^ = 0%, *p* = 0.674 > 0.05] (40–50 years old) and [MD = −4.60, 95%CI (−5.67, −3.53), *p* < 0.05] (age > 50 years old). The results showed that VRT combined with antivertigo could improve daily activities and vestibular function in VN patients. Similarly, sensitivity analysis was used to verify the stability of the meta-analysis, and the results showed that no matter which study was excluded, the combined results of the other studies were not statistically significant [MD = −8.95, 95%CI (−12.39, −5.52)], and the results were stable.

At present, the diagnosis of VN mainly relies on vestibular evoked myogenic potentials (VEMPs) and involved semicircular canal paresis (CP). VEMPs are myoelectric responses from the vestibular labyrinth induced by sound, vibration, or electrical stimulation, and are often used to measure otolith dysfunction (25). Some studies have suggested that the recovery of vestibular nerve injury in VN patients can be judged by observing the dynamic changes of VEMPs (48), and the abnormal number of VEMPs will decrease with the improvement of VN. The results of meta-analysis showed that VRT combined with antivertigo drugs could reduce the abnormal rate of VEMPs [RR = 0.63, 95%CI (0.40, 0.97), *I*^2^ = 0%, *p* = 0.959 > 0.05] and promote the recovery of vestibular function. The semicircular canal is a sensory device in the inner ear associated with maintaining posture and balance. Semicircular canal paresis is caused by nervous system damage, often accompanied by ataxia, balance dysfunction (49), Ceng believes that CP score can objectively evaluate semicircular canal function. The results of meta-analysis showed that VRT combined with anti-vertigo drugs could improve the BBS score of VN patients [MD = 6.84, 95%CI (6.08, 7.60), *I*^2^ = 38.3%, *p* = 0.166 > 0.05]. CP score was decreased [MD = −6.11, 95%CI (−8.02, −4.21), *I*^2^ = 30.8%, *p* = 0.236 > 0.05], and balance ability and vestibular function of patients were improved to a certain extent.

The etiology and pathogenesis of VN are not fully understood, but previous studies have shown that a variety of factors may be related to its pathogenesis. Firstly, viral infection leading to vestibular neurodegeneration is considered one of the most common causes of VN. There are two main types of viral infection: one is respiratory pathogen, which is seasonal and clustered (50), and the other is dormant HSV-1 virus, which is activated and exists in latent form in the vestibular ganglion of human, eventually leading to vestibular inflammation, and then causing vertigo and other symptoms (6). Studies have shown that vaccinating mice with herpes simplex virus induces vestibular ganglion cells in mice to become infected with VN after vestibular dysfunction. Secondly, the pathological mechanism of VN may be related to the inflammatory process caused by infection. Inflammatory factors such as interleukin-6 (IL-6), tumor necrosis factor-α (TNF-α) and C-reactive protein (CRP) are highly expressed in the body with human herpes virus, which is also related to the herpes virus infection mechanism of VN (51). Thirdly, VN may be caused by vascular lesions of the nerves. Multiple causes of labyrinthine artery stenosis or obstruction occlusion, ischemia and hypoxia of perivestibular organs, leading to rapid unilateral vestibular dysfunction (52). Fourthly, autoimmunity also mediates the occurrence of VN. The marker CD40 in monocytes/macrophages plays an important role in inflammation, vascular processes and immunity in VN (53). The immune imbalance between T-helper and T-suppressor cells is also closely related to VN (54). In addition, other factors such as vitamin D deficiency (55) and metabolic diseases such as diabetes may also contribute to the development of VN (56).

At present, the treatment of VN is mainly based on autonomic symptoms such as vomiting and vertigo and the severity of the disease. The therapeutic mechanism is to improve cerebral blood circulation, through vestibular inhibitors, neuroprotective agents or vestibular rehabilitation training and surgical procedures to improve central compensation (50, 57, 58). Antivertigo drugs are used for vertigo accompanied with nausea and vomiting in patients with acute VN stage. As the mechanism of action of such drugs is to delay the establishment of central compensation and affect the prognosis of VN, they cannot be used for a long time (57). VRT is a kind of non-invasive physical therapy. Its principle of action is realized through the plasticity and compensatory capacity of the vestibular nervous system. The mechanism of action is to readjust eye movement, proprioception and postural control through the reorganization of brain stem and cerebellar pathways, and then achieve the effect of treating vestibular vertigo (59–61). However, single drug therapy or VRT is always difficult to achieve the expected effect, and some studies have found that VRT combined drug therapy can better promote the rehabilitation of VN patients (62). As an important method for the treatment of VN, VRT has the advantages of simplicity, economy, non-invasive, strong compliance, etc. VRT should be used in combination with drug therapy with strong symptomatic and rapid effect, and be widely promoted in clinical practice.

A total of 3 RCTs in the included study reported adverse effects. One study (30) reported mild and transient discomfort, such as indigestion, facial swelling, and mood swings. In Goudakos’ antivertigo trial (25), one patient using hypoglycemic drugs to control diabetes developed disease instability and hyperglycemia, and blood sugar returned to normal levels after dose adjustment. Xu’s study (32) reported 4 cases of general fatigue, 5 cases of insomnia, and 1 case of dryness-heat.

This meta-analysis was conducted by developing strict inclusion/exclusion criteria and controlling for methodological quality, however some limitations remain. Firstly, this meta-analysis strictly followed the inclusion and exclusion criteria for literature screening, but most of the included studies were from China and there were few English studies, which may lead to the existence of country bias. So new meta-analyses are needed after more English studies are published in the future to ensure the comprehensiveness and impartiality of the studies. Secondly, some of the studies in our review have methodological flaws. The most common methodological flaw was a lack of blindness to participants, therapists, and evaluators. Thirdly, some studies have small sample size, short intervention time, and lack of follow-up, so larger and high-quality randomized controlled trials are needed. Finally, according to the inclusion criteria, only five RCTs used BBS as an outcome indicator. Although our conclusion is positive, confirming that VRT combined with antivertigo does promote the restoration of balance function in VN patients, more high-quality studies are needed to verify this conclusion in the future.

## 5. Conclusion

VRT combined with antivertigo drugs can improve vertigo and balance function in VN patients. At the same time, combined therapy can also enhance vestibular nerve and muscle function of VN patients, promote the recovery of otolith dysfunction, reduce the impact of vertigo on daily activities, and improve the quality of life of patients. In addition, the combination treatment had fewer adverse effects, demonstrating safety. However, there are shortcomings such as small sample size, short intervention time, and lack of long-term follow-up. In the future, larger sample size and higher quality randomized controlled trials are needed to further verify the effectiveness of VRT combined with anti-vertigo drugs on VN. A new meta-analysis could determine which class of drugs VRT in combination is more effective in treating VN.

## Author contributions

JC: Writing – original draft, Writing – review & editing. ZXL: Writing – review & editing. YLX: Writing – review & editing. SJ: Funding acquisition, Writing – review & editing.
